# The association between low-dose aspirin intake and osteoarthritis: a population-based cross-sectional study based on NHANES

**DOI:** 10.3389/fmed.2024.1413174

**Published:** 2024-10-09

**Authors:** Binglang Xiong, Cheng Zhang, Xuhan Cao, Ziyan Guo, Zixing Bai, Weidong Sun

**Affiliations:** ^1^Second Department of Orthopedics, Wangjing Hospital of China Academy of Chinese Medical Sciences, Beijing, China; ^2^Third Department of Orthopedics, Wangjing Hospital of China Academy of Chinese Medical Sciences, Beijing, China; ^3^Department of Orthopedics, Shunyi Hospital, Beijing Traditional Chinese Medicine Hospital, Beijing, China

**Keywords:** aspirin, osteoarthritis, prevalence, NHANES, cross-sectional study

## Abstract

**Objectives:**

Low-dose aspirin is widely used as a preventive medication for cardiovascular diseases. However, there is controversy regarding the impact of low-dose aspirin on articular cartilage. The aim of this study is to explore the association between low-dose aspirin intake and osteoarthritis (OA).

**Methods:**

We conducted a cross-sectional study based on the United States population data from the National Health and Nutrition Examination Survey (NHANES) 2011–2018. The investigation of low-dose aspirin intake and the diagnosis of OA was based on self-reporting in questionnaires. Multivariate regression models was used to assess the relationship between low-dose aspirin intake and OA. In addition, subgroup and interaction analysis were performed to assess the robustness of the results.

**Results:**

A total of 12,215 participants were included in this study. Multivariate logistic regression analysis showed that low-dose aspirin use had significantly increased the odds of OA (OR = 1.14; 95% CI: 1.01–1.28; *p* = 0.035). A significant and consistent association of low-dose aspirin intake with OA was still observed in each subgroup stratified by gender, age, and the presence of comorbidities including diabetes, coronary heart disease, hypertension, and stroke. The results illustrated that the relationship between low-dose aspirin intake and OA was stable in all subgroups and had no interaction.

**Conclusion:**

Our study confirmed that low-dose aspirin intake may increase the risk of OA. Attention should be paid to the possibility of joint degenerative changes in patients who take low-dose aspirin chronically. However, further studies are needed to explore the possible mechanisms behind this association.

## Introduction

Osteoarthritis (OA) is the most common degenerative joint disease globally, affecting ~500 million people over 40 years old (1). OA often leads to joint stiffness and deformity, accompanied by chronic pain and joint mobility dysfunction, seriously affecting the quality of life (2). Among the 369 diseases assessed in the Global Burden of Disease study, OA ranks 18th in terms of disability rate (3). The occurrence of OA is the result of multiple factors, including age, gender, obesity, genetics, diet, injury, joint dislocation and abnormal joint loading (4, 5). Currently, treatment for OA only relieves pain or controls symptoms, but cannot reverse the progression of the disease (6). Patients with severe OA need to be treated with artificial joint replacement, but it comes with a substantial financial cost (7, 8). Therefore, early identification of risk and protective factors for the development of OA is essential to slow down the progression of the disease.

Aspirin is one of the most widely used drugs in the world today, with various efficacies including antipyretic, analgesic, and anti-inflammatory properties (9). Because aspirin irreversibly inhibits cyclooxygenase, thereby reducing platelet aggregation, it is most commonly used to prevent complications associated with atherothrombosis (10, 11). In recent years, aspirin has been applied in the clinical treatment of other diseases due to its wide range of effects, and these applications are unrelated to its original use (12). In addition, the function of aspirin as an anticoagulant makes it useful in the prevention and treatment of preeclampsia (13). Due to its anti-inflammatory properties, daily low-dose aspirin intake has been shown to reduce colon cancer incidence and mortality (14). Therefore, aspirin is also considered as a miracle drug (15).

However, the effect of aspirin on articular cartilage remains unclear. A recent experimental study found that aspirin alleviates the inhibitory effect of TNF-α on chondrogenesis of Bone marrow mesenchymal stem cells (BMMSCs) by stabilizing Yes-associated protein, and reduces the progression of cartilage degeneration in a mouse model of medial meniscus (16). This contradicts the previous view that aspirin can cause OA-like changes in normal cartilage and may worsen the OA disease process (17). In the United States, more than 30% of adults take low-dose aspirin daily for cardiovascular disease prevention (18). Thus, exploring whether low-dose aspirin is related to the development of OA may provide assistance in the prevention of OA.

Few clinical studies have evaluated the effects of low-dose aspirin intake on OA. So, we conducted this cross-sectional study using data from the National Health and Nutrition Examination Survey (NHANES) 2011–2018. The aim is to investigate the association between daily low-dose aspirin intake and OA in the adult population aged 40 and above in the United States.

## Materials and methods

### Study population

**Figure 1:**
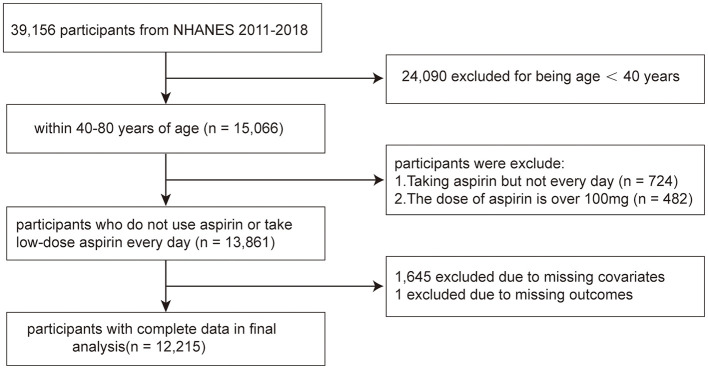
The flowchart of participants.

The NHANES is a population-based cross-sectional survey and research program conducted by the Centers for Disease Control and Prevention (CDC) in the United States to assess the health and nutritional status of adults and children in the country. Starting from the 1980s, this database has continuously collected information about the health and nutrition of the American household population, including demographic, dietary, examination, laboratory, questionnaire, and limited access data. The survey was approved by the Research Ethics Review Committee of the CDC, which was approved by all adult participants in written informed consent. Data from 2011 to 2018 in NHANES were combined in this study. In total, 15,066 participants aged over 40 years were included. Seven hundred and twenty-four participants were excluded because they took aspirin but not on a regular daily basis, and 482 participants were not included for they took doses >100 mg. After excluding samples with missing value for OA status information (*n* = 1) and other covariates (*n* = 1,645), 12,215 participants were enrolled for analysis (Figure 1).

### Definition of low-dose aspirin intake

The definition of low-dose Aspirin Intake in this study were based on in-person home interviews with participants who reported aspirin use prophylaxis by their own or followed recommendations for low-dose aspirin use. Participants were asked “Taking low-dose aspirin on your own?” and “Followed advice, took low-dose aspirin?” Those who answered “Yes” were defined as aspirin Intake. If the answer was “yes”, the patients will be further to answer follow-up questions, “frequency of aspirin use?”. The answers included “One every other day, another schedule, refused, don't know” were excluded from the statistical analysis. Finally, we used ≤ 100 mg/d as low-dose aspirin Intake in accordance with prior studies and recommendations (19, 20).

### Diagnosis of OA status

Diagnosis of OA was mainly based on questionnaires. Participants were asked two osteoarthritis-related questions in succession. Firstly, when asked “Has a doctor ever told you that you had arthritis?”, if the answer is “yes”, the participant would be further asked “Which type of arthritis was it?”. Then, participants who answered “Osteoarthritis or degenerative arthritis” were identified as having OA.

### Covariates

Other covariates that could confound the association between Low-dose Aspirin Intake and OA were included. Socio-demo graphic covariates included age, gender, body mass index (BMI), race (including Mexican American, Non-Hispanic White, Non-Hispanic Black, and others), education level (including lower than high school, completed high school, and more than high school) and poverty-to-income ratio (PIR). PIR < 1 was deemed as poor, while PIR ≥ 1 was regarded as not poor. Physical activity was assessed using the Physical Activity Questionnaire (PAQ) that is embedded in the NHANES survey, and was categorized as inactive, moderate, vigorous and both moderate and vigorous. Smoking at least 100 cigarettes per year was defined as being a smoker. Consuming alcohol more than 12 times a year was considered as being a drinker. Hypertension was defined as having a mean blood pressure value of at least 130 mmHg for systolic blood pressure or at least 80 mmHg for diastolic blood pressure, based on three measurements (21). The diagnosis of diabetes was primarily based on whether the participant had ever been informed by a doctor of having diabetes, or had a glycated hemoglobin level >6.5%, or a fasting blood glucose level >7.0 mmol/L, or was currently using insulin or other hypoglycemic medications. A history of coronary heart disease and stroke depends on whether the patient has been diagnosed by a doctor. Other covariates include total cholesterol, triglyceride, and high-density lipoprotein (HDL).

### Statistical analysis

Continuous variables were expressed as mean ± standard deviation, while categorical variables were represented as percentages. We categorized the participants into groups based on low-dose aspirin Intake. We utilized the ANOVA tests to analyze continuous variables exhibiting a normal distribution, whereas the Kruskal–Wallis test was employed for those continuous variables that did not adhere to a normal distribution. The chi-square tests were used to evaluate the characteristics of the participants for categorical variables. Multivariate logistic regression analyses were conducted to assess the association between low-dose aspirin Intake and OA, presenting the results with odds ratios (OR) and their corresponding 95% confidence intervals (CI). Four models were established: Crude model: no covariates were adjusted. Model 1: age, gender, and race were adjusted. Model 2: age, gender, race, body mass index, education level, poverty-to-income ratio, physical activity, smoking status and drinking status were adjusted. Model 3: all covariables are adjusted.

### Ethics approval and consent to participate

The board of the National Center for Health Statistics reviewed and approved the studies that involved human participants, and all participants gave their written informed consent to take part in the study.

## Results

### Characteristics of participants

Table 1 shows the baseline characteristics of participants in the no-aspirin group (*n* = 9,168) and the low-dose aspirin intake group (*n* = 3,047). The low-dose aspirin intake group was older than the no-aspirin group (66.6 ± 10.1 vs. 56.9 ± 11.7, *p* < 0.001). Age stratification showed that in the low-dose aspirin intake group, there were more people aged 60–69 and ≥70 years old, accounting for 33.6 and 42.1%, respectively. The non-aspirin group had a higher proportion of participants aged 40–49 (32.7%) and 50–59 (27.3%). There are also differences in gender proportions between the two groups. In the low-dose aspirin intake group, males account for 52.87% and females account for 47.2%. In the non-aspirin group, males account for 46.0% and females account for 54.0%. No differences were found between the two groups in terms of education level and drinking status. The no-aspirin group had a lower BMI, was more engaged in physical activity, and had a lower smoking rate than the low-dose aspirin intake group. The low-dose aspirin intake group had a higher incidence of OA than the no-aspirin group (22.9 vs. 13.9%). In addition, statistical differences were also found when comparing PIR, smoking status, diabetes, coronary heart disease, stroke, total cholesterol, triglyceride, and HDL between the two groups (*p* < 0.05).

**Table 1:** Baseline characteristics of the study participants.

	**No aspirin use (*N* = 9,168)**	**Low-dose aspirin intake (*N* = 3,047)**	**Total (*N* = 12,215)**	***P*-value**
Age, years	56.9 ± 11.7	66.6 ± 10.1	59.3 ± 12.1	< 0.001
40–49	2,998 (32.7%)	182 (6.0%)	3,180 (26.0%)	
50–59	2,505 (27.3%)	559 (18.3%)	3,064 (25.1%)	
60–69	2,130 (23.2%)	1,024 (33.6%)	3,154 (25.8%)	
≥70	1,535 (16.7%)	1,282 (42.1%)	2,817 (23.1%)	
Gender				< 0.001
Male	4,219 (46.0%)	1,609 (52.8%)	5,828 (47.7%)	
Female	4,949 (54.0%)	1,438 (47.2%)	6,387 (52.3%)	
BMI, kg/m^2^	29.4 ± 6.86	30.4 ± 6.78	29.6 ± 6.85	< 0.001
Race				< 0.001
Mexican American	1,305 (14.2%)	326 (10.7%)	1,631 (13.4%)	
Non-Hispanic White	3,290 (35.9%)	1,384 (45.4%)	4,674 (38.3%)	
Non-Hispanic Black	2,010 (21.9%)	713 (23.4%)	2,723 (22.3%)	
Other	2,563 (28.0%)	624 (20.5%)	3,187 (26.1%)	
Education level				0.902
< High school	1,094 (11.9%)	357 (11.7%)	1,451 (11.9%)	
Completed high school	1,151 (12.6%)	404 (13.3%)	1,555 (12.7%)	
>High school	6,923 (75.5%)	2,286 (75.0%)	9,209 (75.4%)	
PIR				0.0344
Poor	1,664 (18.2%)	490 (16.1%)	2,154 (17.6%)	
Not poor	7,504 (81.9%)	2,557 (83.9%)	10,061 (82.4%)	
Physical activity				0.0164
Inactive	5,780 (63.0%)	1,949 (64.0%)	7,729 (63.3%)	
Moderate	1,758 (19.2%)	643 (21.1%)	2,401 (19.7%)	
Vigorous	390 (4.3%)	106 (3.5%)	496 (4.1%)	
Moderate and vigorous	1,240 (13.5%)	349 (11.5%)	1,589 (13.0%)	
Smoking status				< 0.001
Yes	3,990 (43.5%)	1,532 (50.3%)	5,522 (45.2%)	
No	5,178 (56.5%)	1,515 (49.7%)	6,693 (54.8%)	
Drinking status				0.55
Yes	5,210 (56.8%)	1,697 (55.7%)	6,907 (56.5%)	
No	3,958 (43.2%)	1,350 (44.3%)	5,308 (43.5%)	
Diabetes				< 0.001
No	7,348 (80.1%)	1,710 (56.1%)	9,058 (74.2%)	
Yes	1,820 (19.9%)	1,337 (43.9%)	3,157 (25.8%)	
Coronary heart disease				< 0.001
No	8,974 (97.9%)	2,602 (85.4%)	11,576 (94.8%)	
Yes	194 (2.1%)	445 (14.6%)	639 (5.2%)	
Stroke				< 0.001
No	8,850 (96.5%)	2,732 (89.7%)	11,582 (94.8%)	
Yes	318 (3.5%)	315 (10.3%)	633 (5.2%)	
Total cholesterol, mmol/L	5.14 ± 1.06	4.72 ± 1.14	5.03 ± 1.09	< 0.001
Triglyceride, mmol/L	1.79 ± 1.56	1.84 ± 1.32	1.80 ± 1.50	< 0.001
HDL, mmol/L	1.40 ± 0.43	1.36 ± 0.42	1.39 ± 0.43	< 0.001
Hypertension				< 0.001
No	3,735 (40.7%)	496 (16.3%)	4,231 (34.6%)	
Yes	5,433 (59.3%)	2,551 (83.7%)	7,984 (65.4%)	
Osteoarthritis				< 0.001
No	7,894 (86.1%)	2,350 (77.1%)	10,244 (83.9%)	
Yes	1,274 (13.9%)	697 (22.9%)	1,971 (16.1%)	

### Association between low-dose aspirin intake and OA

We examined the association between low-dose Aspirin Intake and OA in multivariable logistic regression analysis (Table 2). Without adjusting for any covariates, a significant correlation between low-dose aspirin Intake and OA was detected in crude model (OR = 1.84; 95% CI, 1.66–2.04, *p* < 0.001). These associations remained significant after adjustments were made for age, gender, and race in Model 1 (OR = 1.29; 95% CI, 1.15–1.44, *p* < 0.001) and additionally adjusting for BMI, education level, PIR, Physical activity, smoking status and drinking status in model 2 (OR = 1.19; 95% CI, 1.06–1.33, *p* < 0.001). Moreover, our results showed that the association between low-dose Aspirin Intake and OA remained stable and significant even with adjustment for all Covariates in mode 3 (OR = 1.14; 95% CI, 1.01–1.28, *p* = 0.035).

**Table 2:** Association of low-dose aspirin intake with OA among participants in the NHANES 2011–2018 cycle.

**Model**	**No aspirin use**	**Low-dose aspirin (95% Cl)**	***P-*value**
Crude model	Ref	1.84 (1.66, 2.04)	< 0.001
Model 1	Ref	1.29 (1.15, 1.44)	< 0.001
Model 2	Ref	1.19 (1.06, 1.33)	0.003
Model 3	Ref	1.14 (1.01, 1.28)	0.035

Crude model: no covariates were adjusted.

Model 1: age, gender, and race were adjusted.

Model 2: age, gender, race, BMI, education level, PIR, physical activity, smoking status and drinking status were adjusted.

Model 3: age, gender, race, BMI, education level, PIR, physical activity, smoking status, drinking status, diabetes status, coronary heart disease, stroke status, hypertension status, total cholesterol, triglyceride, HDL were adjusted.

Table 3 shows the stratified analysis and interaction tests by gender, age, diabetes, coronary heart disease, stroke and hypertension status. The subgroup analysis revealed effect of low-dose Aspirin Intake on OA was consistent across all subgroups. The interactions with all groups are not statistically significant (*P* for interaction > 0.05).

**Table 3:** Subgroup and interaction analysis of the association of low-dose aspirin intake and OA.

	**No aspirin use**	**Low-dose aspirin**	***P* for interaction**
Gender			0.512
Male	Ref	1.20 (1.00, 1.43), *p* = 0.047	
Female	2.27 (1.97, 2.62), *p* < 0.001	2.52 (2.12, 3.00), *p* < 0.001	
Age			0.205
40–49	Ref	1.54 (0.92, 2.57), *p* = 0.097	
50–59	2.15 (1.76, 2.62), *p* < 0.001	2.98 (2.26, 3.93), *p* < 0.001	
60–69	3.63 (2.98, 4.42), *p* < 0.001	4.11 (3.27, 5.16), *p* < 0.001	
≥70	6.12 (4.99, 7.51), *p* < 0.001	6.35 (5.12, 7.87), *p* < 0.001	
Diabetes			0.105
No	Ref	1.14 (1.00, 1.29), *p* = 0.041	
Yes	1.37 (0.96, 1.95), *p* = 0.082	1.70 (1.35, 2.15), *p* < 0.001	
Coronary heart disease			0.680
No	Ref	1.14 (1.00, 1.29), *p* = 0.042	
Yes	1.37 (0.96, 1.95), *p* = 0.083	1.70 (1.35, 2.15), *p* < 0.001	
Stroke			0.066
No	Ref	1.19 (1.05, 1.34), *p* = 0.007	
Yes	1.32 (0.99, 1.75), *p* = 0.055	1.07 (0.80, 1.43), *p* = 0.647	
Hypertension			0.478
No	Ref	1.05 (0.80, 1.38), *p* = 0.732	
Yes	1.15 (1.00, 1.33), *p* = 0.047	1.35 (1.14, 1.59), *p* < 0.001	

Age, gender, race, BMI, education level, PIR, physical activity, smoking status, drinking status, diabetes status, coronary heart disease, stroke status, hypertension status, total cholesterol, triglyceride, HDL were adjusted (the stratified variable was omitted from the mode).

## Discussion

In this study, we investigated the relationship between low-dose aspirin intake and OA in the US population aged over 40 based on the NHANES database. The results confirmed that low-dose aspirin intake increases the risk of OA. Subgroup analysis revealed no significant difference in results among the various groups.

OA is a slowly progressing disease that primarily affects the elderly, impairs mobility, and increases mortality rate (22). The main pathological characteristics of OA are cartilage degeneration, bone calcification and synovitis. The most prominent feature is the degeneration of hyaline articular cartilage (23). The etiology of OA remains incompletely understood, but recent studies suggest that OA is a disease influenced by multiple pathogenic factors (24, 25). Metabolic diseases, such as obesity, hypertension, dyslipidemia, diabetes and insulin resistance, are considered to be more likely to cause cartilage damage and are related to the development of OA (5). Therefore, we adjusted for metabolic diseases when performing multivariate regression analysis. In addition, inflammation is strongly associated with OA and can affect chondrocyte metabolism during aging (26). Pro-inflammatory cytokine, such as IL-1β and IL-6, are increased in the synovial fluid, synovium, and cartilage of OA (27). Other factors that mediate inflammation, such as NO, PGE2, and cox-2 also have high level in articular cartilage (28, 29).

Aspirin is the prototype drug for non-steroidal anti-inflammatory drugs (NSAIDs). It prevents arachidonic acid from accessing the enzymes by forming an irreversible covalent bond (through acetylation) with the hydroxyl group at serine 530, thereby inhibiting all isoforms of COX (30). Due to its susceptibility to gastrointestinal bleeding, aspirin has been replaced by COX-2 inhibitors in the treatment of fever, pain, and inflammation. Its anti-platelet aggregation effect now makes it more commonly used in low doses to prevent cardiovascular events in high-risk populations (14, 31). Evidence suggests vascular disorders play a role in the pathogenesis of osteoarthritis (32). However, there is controversy over whether aspirin has a protective or toxic effect on articular cartilage and whether it can fundamentally treat OA. The study comparing changes in tibial cartilage volume between aspirin and non-aspirin users concluded that low-dose aspirin (≤ 300 mg/day) reduces medial tibial cartilage defects in patients with KOA for more than 2 years (33). However, the number of participants included in that study was limited and confounding factors were not controlled. The conclusions of this study need to be further confirmed by clinical researches. Based on this we conducted this investigation and the findings are consistent with earlier studies.

The mechanism by which low-dose aspirin intake may increase the risk of OA is still unclear. We have made some speculations on this matter by reviewing previous literature. Chondrocytes are the only cell type present in cartilage, accounting for 1–5% of the cartilage volume. They are embedded in an amorphous extracellular matrix primarily composed of collagen and proteoglycans. Collagen provides tension to the cartilage, while proteoglycans contribute to its compressive resistance (34). After culturing human chondrocytes with aspirin, the synthesis of proteoglycans was significantly reduced (35). An *in vitro* experiment has demonstrated that salicylates can inhibit the synthesis of cartilage proteoglycans by suppressing the activity of Glucuronyltransferase (GT), which is a key enzyme in the synthesis process of chondroitin sulfate (36). According to *in vivo* experimental reports, salicylates can inhibit the biosynthesis of proteoglycans in both normal and degenerate articular cartilage. Additionally, the inhibitory effect is greater on osteoarthritic cartilage compared to normal cartilage (37). Therefore, low-dose aspirin intake by individuals already suffering from osteoarthritis (OA) may exacerbate cartilage damage. Another study comparing the effects of aspirin on cartilage with meloxicam found that acetylsalicylic acid reduced proteoglycan production and cell proliferation, whereas meloxicam did not adversely affect repair processes within the matrix of diseased articular cartilage (38).

Elevated serum uric acid leads to the deposition of urate crystals in the joints, resulting in dysfunction of articular chondrocytes and damage to articular cartilage, similar to endogenous inflammatory processes (39). Daily low-dose aspirin intake (60–300 mg) can reduce uric acid excretion and may induce hyperuricemia, while high doses may lead to uric acid poisoning (40). Therefore, low-dose aspirin intake may increase the risk of OA by elevating uric acid. In addition, we suggest that chronic low-dose aspirin use may persistently exert analgesic effects that tend to diminish pain perception due to joint injury in daily life, causing participants to neglect joint protection, which can lead to the development of occult OA.

Our study has several strengths. The sample size of this study is large enough to provide adequate statistical power, and it allows for the control of a greater number of confounding factors, enabling robust subgroup analysis. However, there are still some limitations. Firstly, this study employs a cross-sectional research design and does not account for the potential impact of the duration of low-dose aspirin use on the incidence of OA. Furthermore, it cannot establish a causal relationship between the two variables. Additional randomized controlled studies are needed to validate these findings. Secondly, since aspirin usage and OA status in this study were primarily obtained through questionnaires administered to participants, there may be a potential for recall bias. Future studies should adopt stricter diagnostic criteria to validate our findings. Finally, even though we adjusted for most confounding variables, there may still be some residual variables that were not accounted for due to the numerous factors that can affect the onset of OA. The genetic background plays an important role in the pathogenesis of OA and The treatment background of patients also affect the efficacy of aspirin. Due to limitations in data availability, our study did not consider their impact on the results, which should be taken into account in future research.

## Conclusion

In summary, our study suggests that low-dose aspirin intake may increase the risk of OA, but further research is needed to prove the specific mechanism behind it.

## Data Availability

The original contributions presented in the study are included in the article/supplementary material, further inquiries can be directed to the corresponding author.
